# Anti-Microbiota Vaccines Modulate the Tick Microbiome in a Taxon-Specific Manner

**DOI:** 10.3389/fimmu.2021.704621

**Published:** 2021-07-12

**Authors:** Lourdes Mateos-Hernández, Dasiel Obregón, Alejandra Wu-Chuang, Jennifer Maye, Jeremie Bornères, Nicolas Versillé, José de la Fuente, Sandra Díaz-Sánchez, Luis G. Bermúdez-Humarán, Edgar Torres-Maravilla, Agustín Estrada-Peña, Adnan Hodžić, Ladislav Šimo, Alejandro Cabezas-Cruz

**Affiliations:** ^1^ Anses, INRAE, Ecole Nationale Vétérinaire d’Alfort, UMR BIPAR, Laboratoire de Santé Animale, Maisons-Alfort, F-94700, France; ^2^ School of Environmental Sciences University of Guelph, Guelph, ON, Canada; ^3^ SEPPIC Paris La Défense, La Garenne Colombes, 92250, France; ^4^ SaBio, Instituto de Investigación en Recursos Cinegéticos (IREC-CSIC-UCLM-JCCM), Ciudad Real, Spain; ^5^ Department of Veterinary Pathobiology, Center for Veterinary Health Sciences, Oklahoma State University, Stillwater, OK, United States; ^6^ Université Paris-Saclay, INRAE, AgroParisTech, Micalis Institute, 78350, Jouy-en-Josas, France; ^7^ Faculty of Veterinary Medicine, University of Zaragoza, Zaragoza, Spain; ^8^ Institute of Parasitology, Department of Pathobiology, University of Veterinary Medicine Vienna, Vienna, Austria

**Keywords:** anti-microbiota vaccines, tick, microbiome modulation, keystone bacteria, networks analysis

## Abstract

The lack of tools for the precise manipulation of the tick microbiome is currently a major limitation to achieve mechanistic insights into the tick microbiome. Anti-tick microbiota vaccines targeting keystone bacteria of the tick microbiota alter tick feeding, but their impact on the taxonomic and functional profiles of the tick microbiome has not been tested. In this study, we immunized a vertebrate host model (*Mus musculus*) with live bacteria vaccines targeting keystone (i.e., *Escherichia-Shigella*) or non-keystone (i.e., *Leuconostoc*) taxa of tick microbiota and tested the impact of bacterial-specific antibodies (Abs) on the structure and function of tick microbiota. We also investigated the effect of these anti-microbiota vaccines on mice gut microbiota composition. Our results showed that the tick microbiota of ticks fed on *Escherichia coli*-immunized mice had reduced *Escherichia-Shigella* abundance and lower species diversity compared to ticks fed on control mice immunized with a mock vaccine. Immunization against keystone bacteria restructured the hierarchy of nodes in co-occurrence networks and reduced the resistance of the bacterial network to taxa removal. High levels of *E. coli*-specific IgM and IgG were negatively correlated with the abundance of *Escherichia-Shigella* in tick microbiota. These effects were not observed when *Leuconostoc* was targeted with vaccination against *Leuconostoc mesenteroides*. Prediction of functional pathways in the tick microbiome using PICRUSt2 revealed that *E. coli* vaccination reduced the abundance of lysine degradation pathway in tick microbiome, a result validated by qPCR. In contrast, the gut microbiome of immunized mice showed no significant alterations in the diversity, composition and abundance of bacterial taxa. Our results demonstrated that anti-tick microbiota vaccines are a safe, specific and an easy-to-use tool for manipulation of vector microbiome. These results guide interventions for the control of tick infestations and pathogen infection/transmission.

## Introduction

Ticks, like many multicellular eukaryotes, harbor a very diverse group of commensal, symbiotic, and pathogenic microorganisms that collectively comprise the microbiome (1, 2). This complex microbial system and the tick have evolved an intimate relationship relevant for tick development, nutritional adaptation, reproductive fitness, ecological plasticity, and immunity (3–6). Mounting evidence shows that non-pathogenic midgut bacteria may also affect tick vector competence and susceptibility to pathogens transmitted by ticks (7–10). The development of high-throughput sequencing technologies and bioinformatics tools in the last decade has significantly improved our knowledge of the phylogenetic and genetic diversity, dynamics, and ecology of the microbial communities in several tick species (2). However, the vast majority of studies are restricted to the analysis of the taxonomic composition of the tick microbiome and except for few of them (11–14), the functional significance of bacterial community structure and diversity remains largely unexplored.

Recent functional metagenomics studies have shown that exploring the taxonomic composition and variability of the tick microbiome underestimates the multidimensional nature of the tick hologenome and that analysis solely based on taxonomic profiles has limited biological significance (11–14). The native tick microbiome is likely composed of bacteria, archaea, fungi, protozoans and viruses with diverse functional capacities, which are engaged in a complex network of cooperative and competitive interactions (1, 2). Some of these microorganisms, known as keystone species, co-occur with many others and may have a large regulatory effect on the structure, organization, and function of the tick microbiome. The ubiquitousness of the keystone taxa is likely associated with important resources they provide to the overall microbial community and/or the tick host (11, 12, 14). This suggests that keystones are an essential component of the functional networks and therefore represent ideal targets for the rational manipulation of the microbial composition and function. The functional capacity of the tick microbiome is not equal to the overall number of its individual components, as microbial species strongly and frequently interact with one another and form a complex functional network (13, 14), which can thus be considered as a fundamental unit in microbial communities of ticks.

Understanding the microbe-microbe relationships is a critical step for predicting their holistic consequences on tick physiology and vector competence (13–15). Microbial co-occurrence networks represent a useful approach to measure the keystoneness of taxa and infer potential interactions between nodes of the functional networks (15, 16). In a recent study, Mateos-Hernández et al. (15, 17) introduced anti-tick microbiota vaccines as a tool to target tick microbiota bacteria. The bacterial family Enterobacteriaceae was identified as a keystone taxon in the microbiome of *Ixodes ricinus* and *Ixodes scapularis*. Immunization with the Enterobacteriaceae bacterium *Escherichia coli* elicited an anti-*E. coli* IgM and IgG antibody (Ab) response associated with increased engorgement weight of *I. ricinus* nymphs that fed on C57BL/6 mice and high mortality in ticks that fed on α-1,3-galactosyltransferase (*α1,3GT*)-deficient C57BL/6 mice compared with the ticks that fed on the control group. High mortality in *I. ricinus* concurred with a wide distribution of genes encoding *α1,3GT* genes in the microbiota of *I. ricinus* and the host’s immune response to α-Gal, a glycan synthetized by bacterial α1,3GT enzymes. The results suggested that tick microbiome disturbance by immune targeting of keystone bacteria and/or bacterial surface molecules such as α-Gal altered the tick-microbiome homeostasis and impacted tick feeding and survival.

In this study, we investigated the impact of immunization with keystone bacteria on the taxonomic and functional profiles and the structure of the microbial communities associated with the tick microbiome. Our findings showed for the first time that host immunization with keystone bacteria is a promising tool for the manipulation of the tick microbiome with the potential to reveal functional mechanisms of the tick-microbiota interactions and spur new strategies to control ticks and tick-borne pathogens.

## Materials and Methods

### Ethics Statement

All procedures were performed at the Animal Facility of the Laboratory for Animal Health of the French Agency for Food, Environmental and Occupational Health & Safety (ANSES), Maisons-Alfort, France, according to French and International Guiding Principles for Biomedical Research Involving Animals (2012). The procedures were reviewed and approved by the Ethics Committee (ComEth, Anses/ENVA/UPEC), with permit number E 94 046 08.

### Mice and Housing Conditions

Six-week-old C57BL/6 (Charles River strain code 027) mice were purchased from Charles River (Miserey, France) and maintained in Green line ventilated racks (Tecniplast, Hohenpeissenberg, Germany) at -20 Pa, with food (Kliba nafaj, Rinaustrasse, Switzerland) and water *ad libitum*. The mice were kept at controlled room temperature (RT, 20-23°C) and a 12-hour (h) light: 12-h dark photoperiod regimen. The animals were monitored twice a day (d) by experienced technicians and deviations from normal behaviors or signs of health deterioration were recorded, and reported.

### Bacterial Cultures and Live Bacteria Immunization

Representative bacteria of the keystone genus *Escherichia*-*Shigella* (i.e., *Escherichia coli*) and the non-keystone genus *Leuconostoc* (i.e., *Leuconostoc mesenteroides*) were selected to be included in live bacteria vaccine formulations, aiming to test the impact of host immune response against “keystone” bacteria on tick microbiota composition, stability and functionality, and tick performance. The selection of these bacteria as live vaccines was based on our previous results (15) that showed that the genus *Escherichia*-*Shigella* of the family Enterobacteriaceae was among the top keystone taxa (i.e., high relative abundance, ubiquitousness, and eigencentrality) identified in *Ixodes* microbiota. Based on the same study (15), we selected the genus *Leuconostoc* of the family Leuconostocaceae as non-keystone bacteria with low keystoneness (i.e., low relative abundance, ubiquitousness, and eigencentrality) in the microbiome of *Ixodes*.

The Gram-negative bacterium *E. coli* BL21 (DE3, Invitrogen, Carlsbad, CA, USA) was prepared as previously described (15). Briefly, *E. coli* was grown on Luria Broth (LB, Sigma-Aldrich, St. Louis, MO, USA) at 37°C under vigorous agitation, washed with phosphate buffer saline (PBS) 10 mM NaH_2_PO_4_, 2.68 mM KCl, 140 mM NaCl, pH 7.2 (Thermo Scientific, Waltham, MA, USA), resuspended at 3.6 × 10^4^ colony-forming unit (CFU)/mL, and homogenized using a glass homogenizer. The gram-positive bacterium *L. mesenteroides* (strain LBH1148, INRAE collection) was grown on MRS broth (Difco, Bordeaux, France) at 37°C without agitation and resuspended and homogenized following the same procedures as for *E. coli*. Six-week-old, C57BL/6 mice were immunized subcutaneously with either *E. coli* (4, 1 × 10^6^ CFU per mouse) or *L. mesenteroides* (4, 1 × 10^6^ CFU per mouse) in a water-in-oil emulsion containing 70% Montanide™ ISA 71 VG adjuvant (Seppic, Paris, France), with a booster dose two weeks after the first dose. Control, C57BL/6 (n = 4) mice received a mock vaccine containing PBS and adjuvant.

### Bacterial Protein Extraction

*Escherichia coli* and *L. mesenteroides* were washed twice with PBS, centrifuged at 1000× g for 5 min at 4°C, resuspended in 1% Trion-PBS lysis buffer (Sigma-Aldrich, St. Louis, MO, USA) and homogenized with 20 strokes using a glass balls homogenizer. The homogenate was then centrifuged at 300× g for 5 min at 4°C and the supernatant was collected. Protein concentration was determined using the Bradford Protein Assay (Thermo Scientific, San Jose, CA, USA) with Bovine Serum Albumin (BSA) as standard.

### Mouse Feces and Sera Sample Collection

Fecal and blood samples were collected on sterile tubes. Blood samples were collected on d0, d14, d30 and d46 in animals from all experimental groups and were incubated for 2h at RT, without anticoagulant, allowing for clotting, and then centrifuged at 5000× g for 5 min at RT, twice. Fresh feces were collected from each animal on d0, d30 and d46. Fecal samples were stored in sterile tubes at −20°C before genomic DNA extraction.

### Indirect ELISA

The levels of Abs reactive against bacterial proteins were measured in mice sera as previously reported (15). The 96-well ELISA plates (Thermo Scientific, Waltham, MA, USA) were coated with 0.5 µg/mL (100 µL/well) of *E. coli* or *L. mesenteroides* protein extracts and incubated for 2 h with 100 rpm shaking at RT. Subsequently, plates were incubated overnight at 4°C. The antigens were diluted in carbonate/bicarbonate buffer (0.05 M, pH 9.6) and incubated overnight at 4°C. Wells were washed three times with 100 µL of PBS containing 0.05% (vol/vol) Tween 20 (PBST), and then blocked by adding 100 µL of 1% Human Serum Albumin (HSA)/PBS for 1 h at RT and 100 rpm shaking. After three washes, sera samples, diluted 1:50 in 0.5% HSA/PBS, were added to the wells and incubated for 1 h at 37°C with shaking. The plates were washed three times and HRP-conjugated Abs (goat anti-mice IgG and IgM) (Sigma-Aldrich, St. Louis, MO, USA) were added at 1:1500 dilution in 0.5% HSA/PBST (100 µL/well) and incubated for 1 h at RT with shaking. The plates were washed three times and the reaction was developed with 100 µL ready-to-use TMB solution (Promega, Madison, WI, USA) at RT for 20 min in the dark, and then stopped with 50 µL of 0.5 M H_2_SO_4_. Optimal antigen concentration and dilutions of sera and conjugate were defined using a titration assay. The optical density (OD) was measured at 450 nm using an ELISA plate reader (Filter-Max F5, Molecular Devices, San Jose, CA, USA). All samples were tested in triplicate and the average value of three blanks (no Abs) was subtracted from the reads. The cut-off was determined as two times the mean OD value of the blank controls.

### Immunofluorescence

*Escherichia coli* and *L. mesenteroides* were washed three times with PBS, centrifuged at 1000x g for 5 min, fixed with 4% paraformaldehyde for 30 min and blocked with 1% human serum albumin (HSA, w/v in PBS) for 1h at RT. Bacterial cells were then incubated for two days at 4°C with pooled sera (from all vaccinated mice, d30) of mice immunized either against *E. coli*, *L. mesenteroides* or the mock vaccine at a dilution of 1:20 (v/v in PBS). Thereafter, bacteria were washed three times with PBS followed by incubation with Alexa Fluor 488 conjugates anti-mouse antibody (Ab) against IgM (Life technologies, Eugene, OR, USA; A21042) and IgG (Life technologies, Eugene, OR, USA; A11029) at a dilution of 1:1000 (v/v in 1% HSA) for 3h at RT. After washing with PBS, bacteria were stained with 2µg/µL of 4’,6-diamidino-2-phenylindole (DAPI) and mounted in ProLong Diamond Antifade (Life Technologies, Eugene, OR, USA; P36961). Image acquisition was performed using a Leica confocal microscope (Leica, Wetzlar, Germany) with 63X oil immersion objective. Representative pictures were assembled in Adobe Illustrator and fluorescence was slightly enhanced using Adobe Photoshop CS6 (Adobe System Incorporated, California, USA).

### Tick Infestation

Unfed *I. ricinus* nymphs were obtained from the colonies of UMR-BIPAR, Maisons-Alfort, France. We used previously validated protocol for *I. ricinus* nymphal stages feeding on mouse (17). Briefly, the mice were anesthetized by isoflurane and the 2 cm outer diameter EVA-foam capsule (Cosplay Shop, Brugge, Belgium) was glued on their shaved backs using a non-irritating latex glue (Tear Mender, USA). Each mouse was infested with twenty *I. ricinus* nymphs on study d40. The individual ticks were deposited, one by one, to the capsule by forceps *via* a slit in the plastic lid that close the capsule. The ticks feeding were visually monitored twice a day.

### DNA Extraction and 16S rRNA Sequencing

Genomic DNA was extracted from unfed nymphs and fully-engorged nymphs. Before DNA extraction, nymphs were washed two times in miliQ sterile water and one time in 70% ethanol. Ticks were pooled (5 ticks per pool) and crushed with glass beads using a Precellys24 Dual homogenizer (Bertin Technologies, Paris, France) at 5500× g for 20 s. Genomic DNA was also extracted from mouse fecal samples in *E. coli*-immunized and mock-immunized mice. Tick and fecal genomic DNA was extracted using a Nucleospin tissue DNA extraction Kit (Macherey-Nagel, Hoerdt, France). Each DNA sample was eluted in 100 µl of sterile water. Genomic DNA quality (OD260/280 between 1.8 –2.0) was measured with NanoDrop™ One (Thermo Scientific, Waltham, MA, USA). More than 900ng of DNA at ≥ 20 ng/μL concentration were send for amplicon sequencing of the bacterial 16S rRNA gene, which was commissioned to Novogene Bioinformatics Technology Co. (London, UK). Libraries were prepared with NEBNext^®^ Ultra™ IIDNA Library Prep Kit (New England Biolabs, MA, USA). A single lane of Illumina MiSeq system was used to generate 251-base paired-end reads from the V4 variable region of the 16S rRNA gene using barcoded universal primers (515F/806R) in samples from unfed nymphs (n = 6 tick pools), nymphs engorged on *E. coli*-immunized (n = 9), *L. mesenteroides*-immunized (n = 5), or mock-immunized (n = 7) mice. Mouse fecal samples from *E. coli*-immunized (n = 12, 4 samples per group per time point, d0, d30 and d46) and mock-immunized (n = 12) mice were sequenced as described above. The raw 16S rRNA sequences obtained from tick samples, and mice fecal samples were deposited at the SRA repository (Bioproject No. PRJNA725498). Two extraction reagent controls were set in which the different DNA extraction steps were performed using the same conditions as for the samples but using water as template. DNA amplification was then performed on the extraction control in the same conditions as for any other sample.

### 16S rRNA Sequences Processing

The analysis of 16S rRNA sequences was performed using QIIME 2 pipeline (v. 2019.1) (18). The sequences in the fastq files were denoised and merged using the DADA2 software (19) as implemented in QIIME 2. The amplicon sequence variants (ASVs) were aligned with q2-alignment of MAFFT (20) and used to construct a phylogeny with q2-phylogeny of FastTree 2 (21). Taxonomy was assigned to ASVs using a classify-sklearn naïve Bayes taxonomic classifier (22) based on SILVA database (release 132) (23). Only the target sequence fragments were used in the classifier (i.e., classifier trained with the primers) (24, 25). Taxa that persisted across serial fractions of the samples using QIIME 2 plugin feature-table (core-features) were considered ubiquitous (18).

### Relative Quantification of Bacterial Genes *atoB* and *eutD* by qPCR

To quantify the relative abundance of *E. coli atoB* and *eutD* genes in the tick microbiome, a qPCR was carried out using primers for *atoB* (atoB-F ‘AAGCACGCTCTGGTTATCGT’ and atoB-R ‘AATGTAGCGCCAGTTCATCC’) *eutD* (eutD-F ‘GGTGGATGGCGAGTTACAGT’ and eutD-R ‘CACGCTACAACCACGAGAGA’) designed using Primer-BLAST. Gene amplification was performed with SYBR Green LightCycler 480 Master mix (Roche, Meylan, France) and the conditions as follow: 50°C for 2 min, 95°C for 10 min, 40 cycles of 95°C for 15 s and 60°C for 1 min. The CT values were recorded, and the relative levels of bacterial DNA were normalized against tick *rsp4* as housekeeping gene. Fold change in relative quantities were calculated using the 2^−ΔΔCt^ ratio method.

### Bacterial Co-Occurrence Networks, Identification of Keystone Taxa and Attack Tolerance Test

Co-occurrence networks were inferred for each dataset, based on taxonomic profiles, collapsed at the genus level. Correlation matrices were calculated using the SparCC method (26), implemented in the R environment. The topological parameters, i.e., the number of nodes and edges, weighted degree, centrality metrics and the hub-score of each node, the diameter of the network, modularity, and clustering coefficient were calculated for each network. Network calculations and visualizations were prepared with the software Gephi 0.9.2 (27). Three criteria were used to identify keystone nodes within the networks as previously described (15): (*i*) high eigenvector centrality values, (*ii*) ubiquitousness, and (*iii*) the combination of high relative abundance and eigenvector centrality values. The resistance of these taxonomic networks to taxa removal (i.e., attack tolerance) was tested on these taxonomic networks. The purpose was to measure their resistance to the systematic removal of nodes, either by a random attack with 100 iterations, or by a directed attack, removing the nodes according to its value of betweenness centrality (the highest, the first). The analysis of the network resistance was done with the package NetSwan for R (28).

### Prediction of Functional Traits in the Tick Microbiome

The 16S rRNA amplicon sequences from each data set were used to predict the metabolic profiling of each sample. PICRUSt2 (29) was used to predict the metagenomes from 16S rRNA amplicon sequences. Briefly, the AVSs were placed into a reference tree (NSTI cut-off value of 2) contained 20,000 full 16S rRNA sequences from prokaryotic genomes, which is then used to predict individual gene family copy numbers for each AVS. The predictions are based on Kyoto Encyclopedia of Genes and Genomes (KEGG) orthologs (KO) (30). The output of these analyses included pathways and EC (Enzyme Commission number) profiling; the pathways were constructed based on the MetaCyc database (31).

### Statistical Analysis

Differences in relative Ab levels (i.e., OD) between groups of immunized mice in the different time points were compared using two-way ANOVA with Bonferroni multiple comparison tests applied for individual comparisons. Microbial diversity analyses were carried out on rarefied ASV tables, calculated using the q2-diversity plugins. The alpha diversity (richness and evenness) was explored using Faith’s phylogenetic alpha diversity index (32) and Pielou’s evenness index (33). Differences in α-diversity metric between groups were assessed using Kruskal-Wallis test (alpha= 0.05). Bacterial β-diversity was assessed using the Bray Curtis dissimilarity (34), and compared between groups using the PERMANOVA test. The differential abundant taxa and functional feature (KO genes and pathways) were explored between bacteria- and PBS-immunized mice, the differential features were detected by comparing the log2 fold change (LFC) using the Wald test as implemented in the compositional data analysis method DESeq2 (35).

Correlations between tick microbiota bacteria abundance and mice Abs levels were calculated with the ANOVA-Like Differential Expression (ALDEx2, v. 1.22.0) correlation analysis (aldex.cor function) as implemented in R (v. 4.0.3). Unpaired non-parametric Mann-Whitney U test was used to compare the tick parameters (i.e., time to complete feeding, the weight of engorged ticks and tick mortality) between groups. Two-way ANOVA and Mann-Whitney U test analyses were performed in the GraphPad 5 Prism software (GraphPad Software Inc., San Diego, CA, USA). Differences were considered significant when *p* < 0.05.

## Results

### Vaccination Against Keystone Bacteria Increased Tick Engorgement Weight

**Figure 1:**
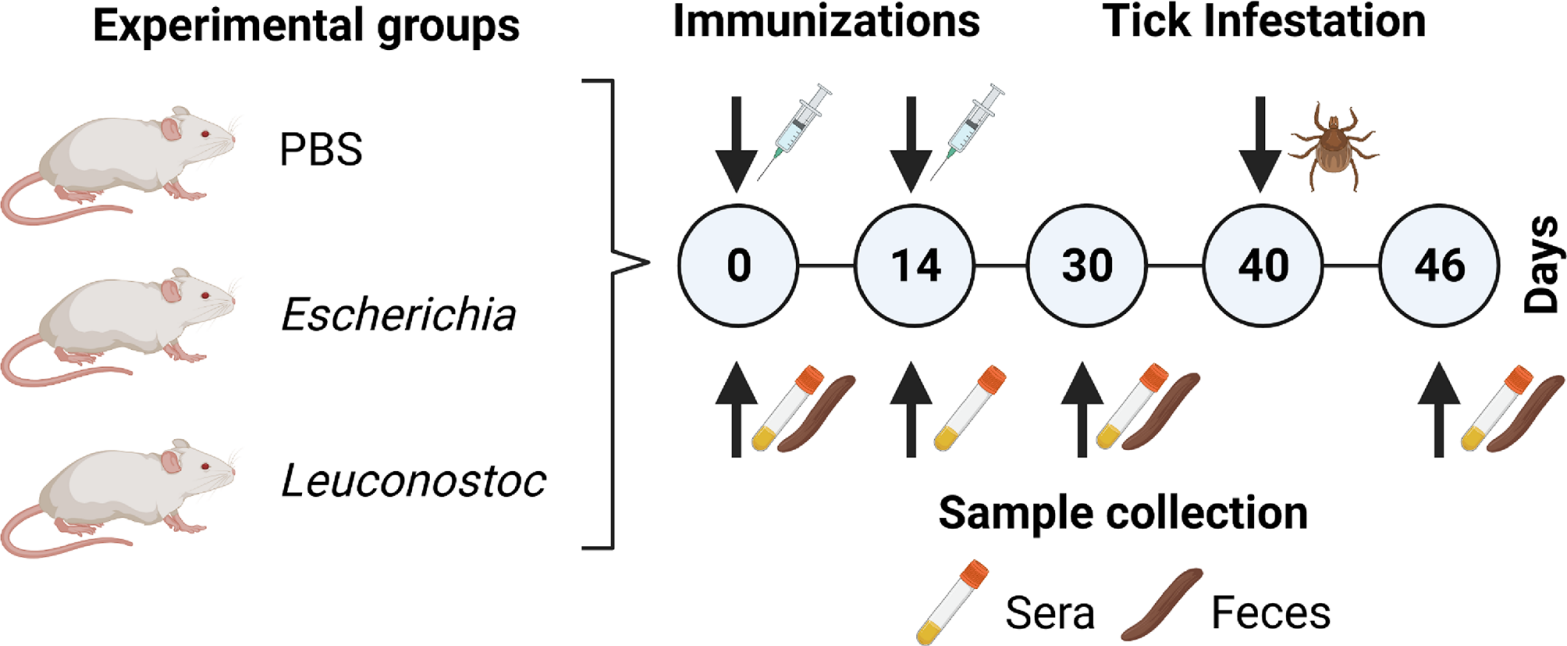
Experimental design and sample collection. Mice were immunized twice (d0 and d14) with a live vaccine containing *E. coli* (n = 4) or *L. mesenteroides* (n = 4) or with a mock vaccine (PBS) (n = 4). At d40, animals in all groups were infested with *I. ricinus* nymphs (n = 20 ticks per mouse). Mice sera (used for ELISA and immunofluorescence) and fecal (used for mice gut microbiota analysis) samples were collected at different time points as indicated. Engorged ticks were collected and used for tick microbiota analysis.

**Figure 2:**
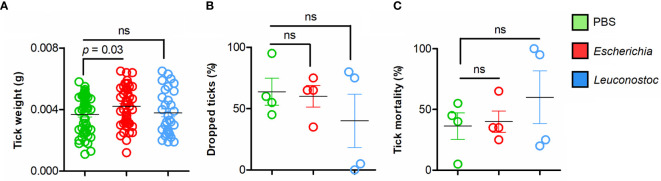
Performance of *I. ricinus* nymphs feeding on mice vaccinated with live *E. coli* or *L. mesenteroides.* **(A)** The weight of individual engorged ticks was measured and compared between groups. **(B)** The percentage of ticks that engorged and dropped was calculated and compared between groups. **(C)** The percentage of dead ticks was calculated and tick mortality (%) was compared between groups. Means and standard deviation values are displayed. The parameters were compared between groups by the Mann-Whitney U test. (ns-not significant; n = 12 mice, n = 20 ticks per mouse).

Following the immunization protocol, each mouse was infested with 20 *I. ricinus* nymphs (**Figure 1**). Time to complete feeding, weight of engorged ticks and tick mortality were recorded and compared between bacteria-immunized groups and control group (immunized with a mock vaccine). A significant increase (Mann-Whitney U test, *p* = 0.03) in weight was recorded in nymphs fed on *E. coli*-immunized mice compared with nymphs fed on mice of the control group (**Figure 2A**). This was not the case for ticks engorged on *L. mesenteroides*-immunized mice (Mann-Whitney U test, *p* > 0.05). There were no significant differences (Mann-Whitney U test, *p* > 0.05) in the total number of ticks that dropped naturally (**Figure 2B**) or the mortality of ticks (**Figure 2C**) that fed on *E. coli*-immunized or *L. mesenteroides*-immunized mice compared with the control group. No mortality or adverse reactions were observed in mice immunized with *E. coli* or *L. mesenteroides*.

### Vaccination Against Keystone Bacteria Reduced Bacterial Diversity in *I. ricinus* Microbiota, and Does Not Affect Mouse Gut Microbiota

**Figure 3:**
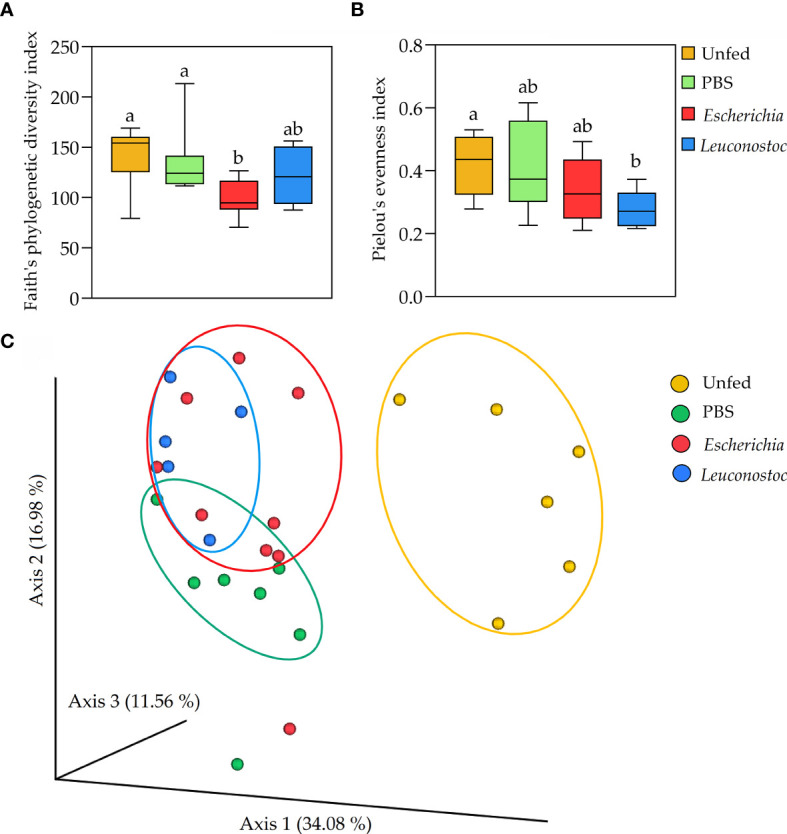
Impact of anti-microbiota vaccines on tick microbial diversity and evenness. **(A)** Faith’s phylogenetic diversity and **(B)** Pielou’s evenness indexes were used to calculated the microbial richness and evenness, respectively, of the bacterial communities of unfed ticks and ticks fed on mock-immunized (green, PBS), *E*. *coli*-immunized (red) and *L. mesenteroides*-immunized (blue) mice. Different letters (‘a’, ‘b’) denote statistical differences between groups. **(C)** First axis PCoA plot showing the variance of taxonomical profile (Bry Curtis distance) at the level of ASVs between samples according to tick feeding status and feeding on immunized mice (PERMANOVA, *p* = 0.00), arbitrary ellipses were drawn to facilitate the interpretation of the figure. The alpha diversity indexes were calculated using 16S rRNA sequences obtained from samples of unfed *I*. *ricinus* nymphs (n = 6 tick pools), and nymphs engorged on *E. coli*-immunized (n = 9 tick pools), *L. mesenteroides*-immunized (n = 5 tick pools), or mock-immunized (n = 7 tick pools) mice.

The impact of anti-microbiota vaccines on the diversity, composition and abundance of tick microbiota bacteria was assessed after 16S rRNA amplicon sequencing of DNA extracted from unfed *I. ricinus* nymphs or from nymphs engorged on *E. coli*-immunized, *L. mesenteroides*-immunized, or mock-immunized mice. Vaccination with *E. coli* reduced the bacterial diversity associated with the tick microbiota (H = 8.6, *p* = 0.03, **Figure 3A**), but had no significant impact (H = 5.8; *p* = 0.12) on the species evenness, compared to unfed nymph (**Figure 3B**). Conversely, vaccination with the non-keystone bacterium *L. mesenteroides* had no significant impact on bacterial diversity (**Figure 3A**), but reduced significantly the species evenness (**Figure 3B**). Overall, the comparison of the diversity indexes of unfed and fed ticks revealed that anti-microbiota vaccination interferes with the normal dynamics of tick microbiota, regardless of the keystoneness of the bacteria used in the vaccine formulation. Accordingly, a Principal Coordinates Analysis (PCoA) showed that the profiles of both groups of ticks that fed on bacteria-immunized mice were very similar compared to mock-immunized or unfed ticks (*F*= 5.30; *p* = 0.00, **Figure 3C**).

To rule out a negative effect of anti-microbiota vaccine on host microbiome, we collected mouse feces at different time points (**Figure 1**) and compared mouse gut microbiota before (d0) and after (d30 and d46) vaccination in the *E. coli*-immunized and mock-immunized mice. Results showed non-significant differences in the microbial richness (*p* > 0.05) between the groups in the different time point, as measured by Faith’s phylogenetic index (**Supplementary Figure S1A**). PERMANOVA (Adonis) test revealed that the factor time modified the Bray Curtis dissimilarity index (*F*= 5.49; *p* = 0.001) in both groups. However, no significant differences in the Bray Curtis dissimilarity index (F = 0.67; *p* = 0.67) or in the interaction vaccine/time (*F*= 1.36; *p* = 0.17) were observed between groups (*E. coli*-immunized *vs.* mock-immunized mice) (**Supplementary Figure S1B**). Furthermore, we observed no significant differences in the taxa abundance between groups at each time point (**Supplementary Figure S1C**). Notably, the relative abundance of *Escherichia-Shigella* in the mouse feces was not affected by the anti-microbiota vaccine containing *E. coli* (**Supplementary Figure S1D**).

The PCR amplification of extraction reagent negative controls (see methods) did not produce DNA products. In addition, rarefaction curves showed that there was sufficient number of reads to draw a reliable list of bacterial genera within each tick and mouse sample (**Supplementary Figure S2**).

### *E. coli*-specific Abs Are Associated With Reduced Abundance of the Keystone Taxon *Escherichia*-*Shigella*

**Figure 4:**
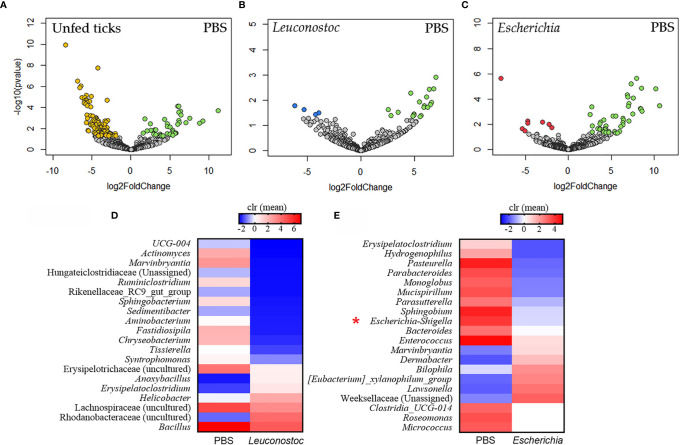
Impact of anti-microbiota vaccines on the taxonomic profiles of tick microbiome. Volcano plot showing differential bacterial abundance in ticks of the different groups: **(A)** unfed ticks *vs.* ticks fed on mock-immunized mice (PBS), **(B)** ticks fed on mock-immunized *vs. L. mesenteroides*-immunized mice, and **(C)** ticks fed on mock-immunized *vs. E. coli*-immunized mice. The yellow (unfed ticks), blue (ticks fed on *L. mesenteroides*-immunized mice), and red (ticks fed on *E*. *coli*-immunized mice) dots indicate taxa that displayed both large magnitude fold-changes and high statistical significance favoring disturbed or control group (green dots), while the gray dots are considered as not significantly different between groups. Relative abundance (calculated as clr transformed values) of the 20 top bacterial taxa with the highest significant differences on ticks fed on mock-immunized *vs. L. mesenteroides*-immunized mice **(D)** and on ticks fed on mock-immunized *vs. E. coli*-immunized mice **(E)**, as detected by the DeSeq2 algorithm (Wald test, *p* < 0.001). *Escherichia*-*Shigella* was marked in the figure (red asterisk). The taxonomic profiles of the tick microbiota were characterized using 16S rRNA sequences obtained from samples of unfed *I*. *ricinus* nymphs (n = 6 tick pools), and nymphs engorged on *E. coli*-immunized (n = 9 tick pools), *L. mesenteroides*-immunized (n = 5 tick pools), or mock-immunized (n = 7 tick pools) mice.

Taxa composition and abundance analysis showed significant changes in the abundance of several bacterial genera in ticks fed on mock-immunized mice compared with unfed ticks (**Figure 4A**). The relative abundance of several taxa changed significantly in the ticks engorged on either *L. mesenteroides*-immunized (**Figure 4B**), or *E. coli*-immunized (**Figure 4C**) mice compared with the control group. The taxa with significant changes in abundance [measured as centered log ratio (clr)] are displayed in **Figures 4D, E** for *L. mesenteroides*-immunized and *E. coli*-immunized mice, respectively. Notably, the abundance of *Escherichia*-*Shigella*, but not *Leuconostoc*, was significantly reduced in ticks that fed on *E. coli*-immunized mice compared with the control group (**Figure 4E**). In contrast, the abundance of *Escherichia*-*Shigella* or *Leuconostoc* was not significantly affected in ticks that fed on *L. mesenteroides*-immunized mice (**Figure 4D**).

**Figure 5:**
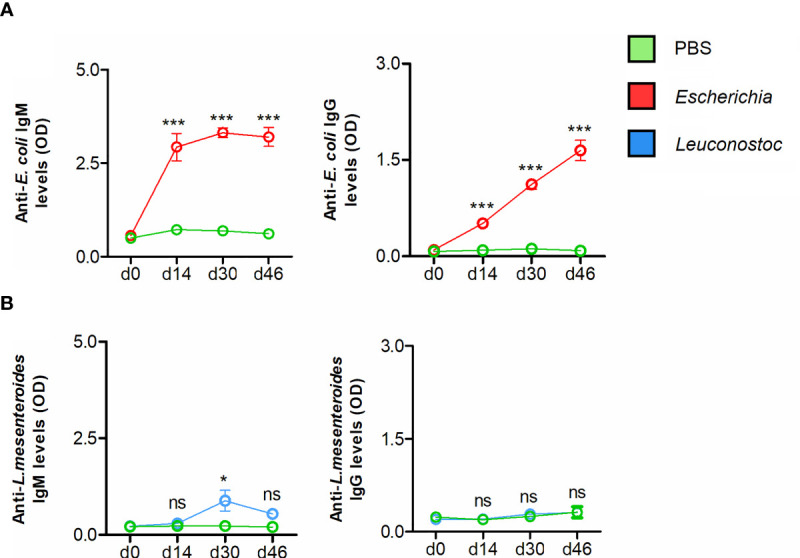
Antibody response of mice vaccinated with live *E. coli* or *L. mesenteroides.* The levels of IgM and IgG specific to **(A)** *E. coli* and **(B)** *L. mesenteroides* proteins were measured by semi-quantitative ELISA in sera of mice immunized with *E. coli* (red) and *L. mesenteroides* (blue), respectively. Antibody levels of bacteria-immunized mice were compared with those of mock-immunized (green, PBS) mice. Means and standard error values are shown. Results were compared by two-way ANOVA with Bonferroni test applied for comparisons between control and immunized mice. (* *p* < 0.05, *** *p* < 0.0001; ns-not significant; 1 experiment, n = 12 mice and three technical replicates per sample).

Immunization with live *E. coli* elicited IgM and IgG responses specific to *E. coli* (**Figure 5A**). Strong and specific immune reaction of mouse IgM against *E. coli* was confirmed by immunofluorescence (**Supplementary Figure S3A**). The immunofluorescence reaction of anti-*E. coli* IgG against *E. coli* was comparatively less intense (**Supplementary Figure S3A**). Immunization with live *L. mesenteroides* elicited only marginal levels of IgM on d30, and the Ab levels had dropped by d46 (**Figure 5B**), suggesting that in contrast to *E. coli*, *L. mesenteroides* was poorly immunogenic as a live vaccine in the conditions used here. Consistent with the low IgM response raised by *L. mesenteroides* immunization, a poor recognition of *L. mesenteroides* by mouse anti- *L. mesenteroides* IgM was observed by immunofluorescence (**Supplementary Figure S3B**). A poor reaction was observed when anti-*L. mesenteroides* IgG detection was used in the immunofluorescence (**Supplementary Figure S3B**), which was consistent with a non-significant increase of anti-*L. mesenteroides* IgG after immunization with live *L. mesenteroides* (**Figure 5B**).

No significant cross-reaction to *E. coli* proteins was detected in the IgM and IgG fractions of the sera of mice immunized with the live vaccine containing *L. mesenteroides* (**Supplementary Figure S4A**). A weak increase in anti-*E. coli* IgM on d30 and d46, and no increase in anti-*E. coli* IgG were observed in *L. mesenteroides*-immunized mice. However, an increase in anti-*L. mesenteroides* IgM (d14, d30 and d46) and IgG (d30 and d46) was detected in response to *E. coli* vaccination (**Supplementary Figure S4B**).

To test a possible association between the increase in *E. coli*-specific IgM and IgG after vaccination and the reduced abundance of *Escherichia*-*Shigella*, we performed an ALDEx2 correlation analysis between the abundance of all bacterial taxa at genus level and Abs levels. Significant negative correlations were found between the levels of anti-*E. coli* IgM (*r*_s_ = -0.60, *p* = 0.01) and IgG (*r*_s_ = -0.57, *p* = 0.02) in mice sera and the abundance of *Escherichia*-*Shigella* in the tick microbiota. Negative correlations between anti-*E. coli* IgM (*r*_s_ = -0.57, *p* = 0.02) and IgG (*r*_s_ = -0.64, *p* = 0.01) levels and an additional bacterial genus (0.18%, total 533 taxa), *Parabacteroides* (Family Porphyromonadaceae) was also found. A positive correlation was found between the genus *Lawsonella* (Order Corynebacteriales), and anti-*E. coli* IgM (*r*_s_ = 0.65, *p* = 0.01) and IgG (*r*_s_ = 0.68, *p* = 0.008) levels. The abundance of no taxa was found to correlate with the levels of both anti-*E. coli* IgM and IgG in the *L. mesenteroides*-immunized mice. In addition, no statistically significant correlations were found between the anti-*L. mesenteroides* IgM and IgG levels and the abundance of *Leuconostoc*, or any other taxa identified in the tick microbiome. Taken together, these results suggest that anti-*E. coli* immunization in mice reduces the *Escherichia*-*Shigella* abundance within the tick microbiome in a taxon-specific manner.

### *Escherichia coli* Vaccination Reduced the Keystoneness of *Escherichia*-*Shigella* and the Tolerance of Co-Occurrence Networks to Taxa Removal

**Figure 6:**
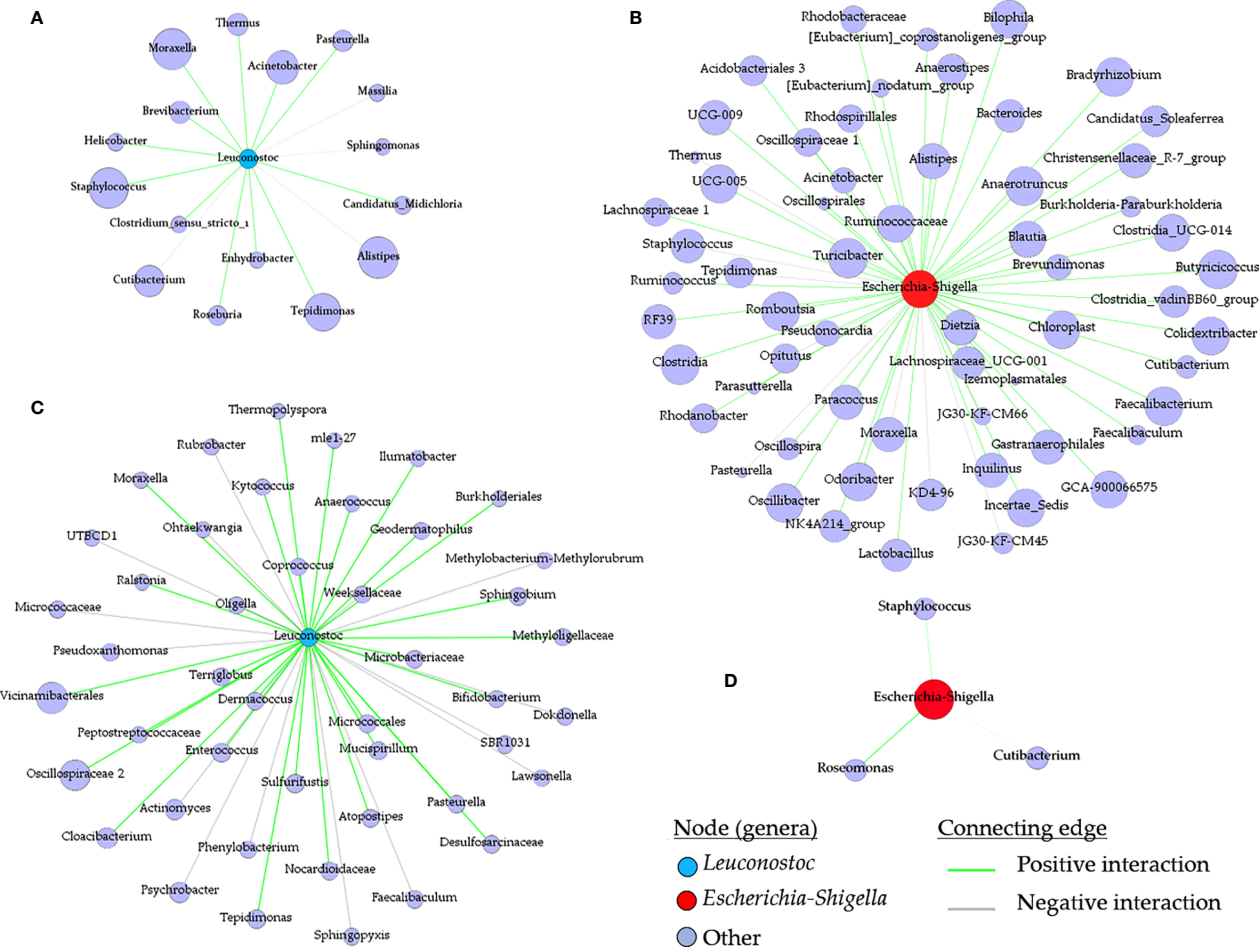
Local connectivity of *Leuconostoc* and *Escherichia*-*Shigella* in the co-occurrence networks. The nodes/taxa linked to *Leuconostoc* (cyan node, **A, C**) and *Escherichia*-*Shigella* (red node, **B, D**) were identified in the bacterial co-occurrence networks of ticks fed on mock-immunized **(A, B)**, *L. mesenteroides*-immunized **(C)** and *E. coli*-immunized **(D)** mice. Connecting edges with positive and negative interactions were also identified.

The impact of live bacteria immunization on the structure of the tick microbial communities was visualized and quantified using co-occurrence networks. In accordance with their classification as non-keystone and keystone taxa, *Leuconostoc* (**Figure 6A**) and *Escherichia*-*Shigella* (**Figure 6B**) had low and high connectivity, respectively, in the microbial community of ticks fed on mock-immunized. Visual inspection of the local connectedness around *Leuconostoc* and *Escherichia*-*Shigella* revealed that the number of co-occurring taxa increased (**Figure 6C**) and decreased (**Figure 6D**) in the networks of tick microbiota exposed to anti-*L. mesenteroides* and anti-*E. coli* Abs, respectively. Notably, the eigenvector centrality value of *Leuconostoc* in the networks inferred from ticks fed on *L. mesenteroides*-immunized mice (eigenvector 0.11) was very similar to that of *Leuconostoc* in the control network (eigenvector 0.12). In contrast, the eigenvector centrality value of *Escherichia*-*Shigella* decreased 95 times in the network of ticks fed on *E. coli*-immunized (eigenvector 0.01) compared with those fed on mock-immunized mice (eigenvector 0.95). Visual (**Supplementary Figure S5**) and numerical (**Table 1**) comparison of the networks showed that, in addition to the local connectedness effect, anti-microbiota vaccination had a large impact on the structure of the microbial community of ticks. For instance, the number of edges in the co-occurring networks of ticks that fed on *E. coli*-immunized and *L. mesenteroides*-immunized mice decreased and increased, respectively, compared with the control network (**Table 1**).

**Table 1:** Topological parameters of co-occurrence networks.

Network features	Control (PBS)	Vaccinated with *L. mesenteroides*	Vaccinated with *E. coli*
Nodes	525	503	518
Edges	2930	7098	910
Positive	2159 (73.7%)	3862 (55.5%)	723 (79.4%)
Negative	771 (26.3%)	3228 (45.5%)	187 (20.6%)
Network Diameter	9	7	10
Average degree	11.2	28.3	8.4
Weighted degree	4.4	2.2	1.7
Average path length	3.4	2.8	3.9
Modularity	1.1	4.9	0.9
Number of modules	22	49	33
Average clustering coefficient	0.6	0.6	0.5

**Figure 7:**
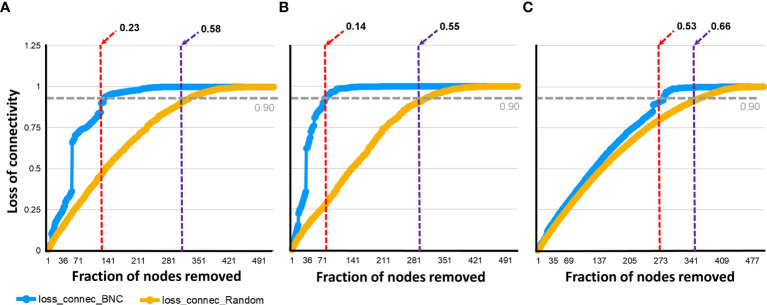
Network tolerance to taxa removal. The resistance of the networks to directed (blue line), or random (orange line), removal of nodes was measured. The proportion of directly (red dashed line), or randomly (violet dashed line) removed nodes that made the network losing 0.90 of connectivity was recorded in the bacterial co- occurrence networks of ticks fed on mock-immunized **(A)**, *L. mesenteroides*-immunized **(B)** and *E. coli*-immunized **(C)**. Loss of connectivity values range between 0 (maximum of connectivity between nodes) and 1 (total disconnection between nodes) for any given network.

Co-occurrence networks were tested for attack tolerance. In this analysis, the resistance of the networks to random or directed removal of nodes was measured and the proportion of taxa removal needed to reach a loss in connectivity of 0.90 was recorded for each network. A proportion of 0.58 randomly removed nodes in the control (**Figure 7A**), 0.55 in the *E. coli* (**Figure 7B**) and 0.66 in the *L. mesenteroides* (**Figure 7C**) networks produced a 0.90 connectivity loss. The same loss in connectivity (i.e., 0.90) was observed when a smaller proportion of highly central nodes, 0.23, 0.14 and 0.53 was removed from the control, *E. coli* and *L. mesenteroides* networks, respectively (**Figure 7**). Thus, immune targeting of the keystone taxon *Escherichia*-*Shigella* decreased the attack tolerance of the bacterial co-occurrence network. The random and directed removal curves within the network of ticks from *L. mesenteroides*-immunized mice revealed high similarity, which was not the case in the other two networks. This result suggests an unstructured hierarchy of nodes in the co-occurrence network of ticks from *L. mesenteroides*-immunized mice, and a reshaping in the hierarchy of nodes in the co-occurrence network of ticks from *E. coli*-immunized mice compared to the control network.

### *Escherichia coli* Vaccination Reduced the Abundance of Lysine Degradation Genes in Tick Microbiome

**Figure 8:**
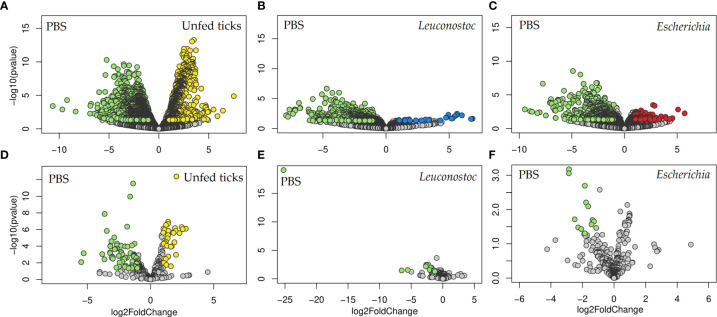
Impact of anti-microbiota vaccines on the predicted functional profiles of tick microbiome. Volcano plot showing differential enzyme **(A–C)** and pathway **(D–F)** abundance in ticks of the different groups: **(A, D)** unfed ticks *vs.* ticks fed on mock-immunized mice (PBS), **(B, E)** ticks fed on mock-immunized *vs. L. mesenteroides*-immunized mice, and **(C, F)** ticks fed on mock-immunized *vs. E. coli*-immunized mice. The yellow (unfed ticks), blue (ticks fed on *L. mesenteroides*-immunized mice), and red (ticks fed on *E. coli*-immunized mice) dots indicate enzyme **(A–C)** and pathway **(D–F)** that displayed both large magnitude fold-changes and high statistical significance favoring disturbed or control PBS group (green dots), while the gray dots are considered as not significant. Differential features were detected by the DeSeq2 algorithm (Wald test, *p* < 0.05). The functional profiles were predicted from 16S rRNA sequences obtained from samples of unfed *I. ricinus* nymphs (n = 6 tick pools), and nymphs engorged on *E. coli*-immunized (n = 9 tick pools), *L. mesenteroides*-immunized (n = 5 tick pools), or mock-immunized (n = 7 tick pools) mice.

We analyzed microbial putative functions displayed by the microbiome of unfed ticks and those that fed on *L. mesenteroides*-immunized and *E. coli*-immunized mice by PICRUSt2 and compared them with the predicted functions of bacterial communities present in the ticks fed on mock-immunized mice (**Figure 8**). Significant differences were found in the relative abundance (fold changes) of several putative genes (KO) in the *I. ricinus* microbiome of nymphs fed on mock-immunized mice compared to unfed ticks (**Figure 8A**, **Supplementary Table S1**). Major changes in the putative gene profiles were also observed in the microbiome of tick from *L. mesenteroides*-immunized (**Figure 8B**, **Supplementary Table S2**) and *E. coli*-immunized (**Figure 8C**, **Supplementary Table S3**) mice.

**Figure 9:**
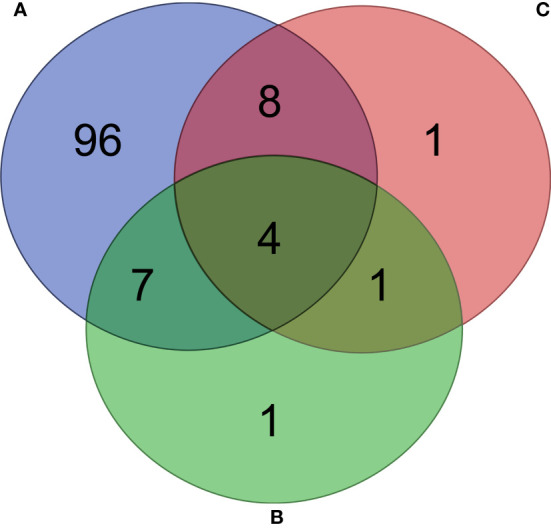
Comparison of unique and share pathways across the different groups. Venn diagram showing the common and different predicted bacterial pathways found in the microbiome of ticks fed on mock-immunized mice compared to unfed ticks **(A)**, and ticks fed on *L. mesenteroides*-immunized **(B)** and *E. coli*-immunized **(C)** mice compared to mock-immunized mice. Only pathways with statistically significant log2 fold changes in the absolute value (cutoff of 1) were considered.

Fold changes in the relative abundance of several pathways were found in unfed ticks (**Figure 8D**), *L. mesenteroides*-immunized (**Figure 8E**) and *E. coli*-immunized (**Figure 8F**) mice, compared to ticks fed on mock-immunized mice. The abundance of 115 pathways changed significantly (81 and 34 with decreased and increased abundance, respectively, Log2fold change > 1, *p* < 0.05) in ticks that fed on mock-immunized mice compared to unfed ticks (**Supplementary Table S4**). Among them, 96 were found exclusively in the microbiome of ticks fed on mock-immunized mice compared to unfed ticks and in none of the vaccinated groups (**Figure 9A**). Four pathways (i.e., methanogenesis from acetate, super pathway of glycerol degradation to 1,3-propanediol, superpathway of (Kdo)2-lipid A biosynthesis, and CMP-legionaminate biosynthesis I) changed significantly in the three groups of fed ticks (**Figure 9**) and they may represent functional changes induced by blood feeding in the bacterial communities independent of the treatment.

Thirteen pathways had a log2fold change lower than -1 in the functional prediction of the microbiome of ticks fed on *L. mesenteroides*-immunized mice, compared to the control group of ticks fed on mock-immunized mice (**Supplementary Table S5**). Only one of them, tetrahydromethanopterin biosynthesis (PWY-6148, Log2fold change = -5.5, Kruskal-Wallis test, *p* = 0.02), changed exclusively in the ticks of the *L. mesenteroides*-immunized group (**Figure 9B**).

**Figure 10:**
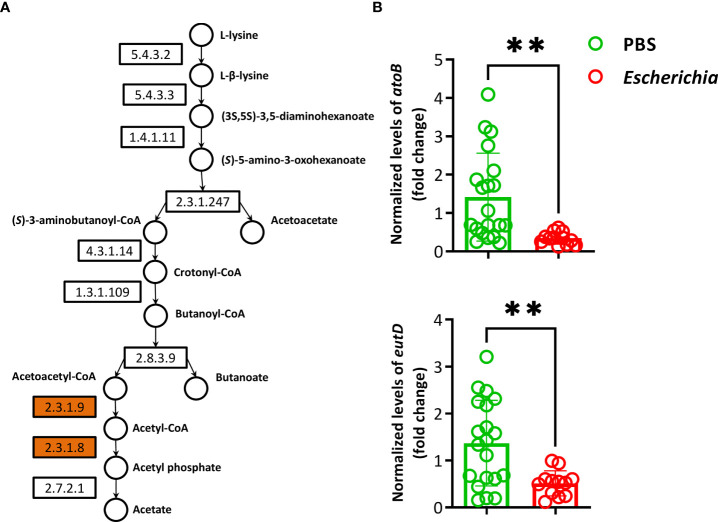
Pathway P163-PWY and relative abundance of genes *atoB* and *eutD*. **(A)** The pathway P163-PWY was retrieved from MetaCyc database. The KEGG codes of the enzymes (boxes) and metabolites (close to the circles) involved in the pathway were added. Enzymes encoded by the genes *atoB* (EC.2.3.1.9) and *eutD* (EC.2.3.1.8) were highlighted (orange). **(B)** Normalized relative *atoB* (EC.2.3.1.9) and *eutD* (EC.2.3.1.8) levels were measured by qPCR in DNA samples of ticks fed on *E. coli*-immunized mice using gene-specific primers and normalizing against tick *rsp4* with the 2^−ΔΔCt^ ratio method. Results are relative to *atoB* and *eutD* in the control group (PBS). Individual technical replicates, means and standard deviation values are displayed. Results were compared by Mann−Whitney U test. (** *p* < 0.001; n = 6 DNA samples of ticks fed on *E. coli*-immunized mice, n = 10 DNA samples of the control group, two technical replicates per samples).

Fourteen pathways had a log2fold change lower than -1 in the functional prediction of the microbiome of ticks fed on *E. coli*-immunized mice, compared to the control group (**Supplementary Table S6**). A significant decrease in the relative abundance of the L-lysine fermentation to acetate and butanoate pathway (P163-PWY, Log2fold change = -1.6, Kruskal-Wallis test, *p* = 0.008) was found exclusively in ticks fed on *E. coli*-immunized mice (**Figure 9C**). The pathway P163-PWY is composed of ten enzymatic steps that transform L-lysine into acetate and butanoate (**Figure 10A**). The bacterial genes *atoB* and *eutD* encoding for the enzymes acetyl-CoA acetyltransferase (EC.2.3.1.9) and phosphate acetyltransferase (EC.2.3.1.8) of the P163-PWY pathway in *E. coli* were selected for validation of the PICRUSt2 prediction. The relative abundance of the genes *atoB* and *eutD* was significantly lower in ticks fed on *E. coli*-immunized mice compared to the control group (**Figure 10B**).

## Discussion

Several studies have shown that the tick microbiome is a gate to access tick physiology and vector competence (1, 36). Reduced bacterial loads have been associated with lower reproductive fitness after antibiotics treatment in ticks (3, 36–40). Considering that antibiotics can target several bacteria species simultaneously, studies using broad-spectrum antimicrobial compounds make impossible establishing causal links between specific taxa and alterations in tick physiology and/or vector competence. Recently, we showed for the first time that anti-tick microbiota vaccines impact tick performance during feeding (15). Immunization with the Enterobacteriaceae bacterium *Escherichia coli* elicited anti-*E. coli* IgM and IgG which were associated with increased engorgement weight of *I. ricinus* nymphs that fed on C57BL/6 mice and high mortality in ticks that fed on α-1,3-galactosyltransferase (*α1,3GT*)-deficient C57BL/6 mice compared with the ticks that fed on the control group, immunized with a mock formulation of PBS and adjuvant (15).

Considering that host Abs and complement acquired during tick feeding not only retain their immune functions, but also access several tick tissues (40–45), we hypothesized that anti-tick microbiota vaccines could be used as a precision microbiology tool to target selected taxa in the tick microbiome. Here, we showed that immunization with a live *E. coli* vaccine elicited bacterial-specific Abs of the isotypes IgM and IgG, which were associated with the reduction of the relative abundance of *Escherichia*-*Shigella* in engorged ticks Therefore, it can be presumed that when ingested during blood feeding, the host Abs can reach the locations of tick microbiota bacteria. Abs induced against particular tick proteins can also reach and react with the corresponding tick protein within tick tissues (43, 46). For example, host Abs against the glycoprotein Bm86, predominantly located in the membrane of tick gut cells (46), modulate tick proteome (47) and bind to the surface of epithelial cells in the tick intestine causing cell lysis (43). The results suggest that host Abs bind and promote the killing of Gram-negative bacteria in the tick microbiota. Upon contact with tick microbiota bacteria, a step-wise activation process may result in the deposition of host complement proteins and insertion of Membrane Attack Complex (MAC) pores into Gram-negative bacterial membranes. This can lead to direct killing of Gram-negative, but not Gram-positive bacteria (48–51). Even though the interaction of polyreactive antibodies with the bacteria surface stimulates the deposition of complement proteins on Gram-positive, this does not result in MAC-mediated lysis of this type of bacteria (48, 51). This suggests that anti-tick microbiota vaccines may be most effective against Gram-negative bacteria. In our study, the live vaccine using the Gram-positive bacterium *L. mesenteroides* had low immunogenicity. Therefore,

Targeting the keystone bacteria *Escherichia*-*Shigella* with host Abs also reduced the keystoneness of this taxon in the networks, which was associated with a global modulation of the microbial community structure. The resulting community had a reduced alpha diversity and changes in the taxonomic and predicted functional profiles. In addition, the co-occurring network analysis showed that *Escherichia*-*Shigella*-depleted communities had fewer nodes and the connectivity between them was weak and more susceptible to taxa extinction when compared with the control group of ticks that fed on mock-immunized mice. Removal of keystone species has strong disturbing effects, resulting in loss of microbiota biodiversity in different ecological settings (52–55). Depletion of keystone species could also result in microbial dysbiosis that impairs the integrity of the gut ecosystem, as seen in vertebrates (56, 57). The impact of *E. coli* removal has been tested experimentally on a synthetic consortium of 14 human gut microbes (57). Removal of *E. coli* resulted in the highest impact on biomass and growth rates, indicating major roles of this microorganism on a synthetic microbial consortium (57).

Here we used PICRUSt2 (29) to predict the metagenomes of tick microbiome using 16S rRNA amplicon sequences. The prediction showed that ticks fed on *E. coli*-immunized mice had a significant reduction in the relative abundance of the pathway P163-PWY. This result was validated by quantifying the abundance of two *E. coli* genes *atoB* and *eutD* encoding for enzymes involved in lysine fermentation *via* P163-PWY. The reduced abundance of these two *E. coli* genes concurs with the reduced abundance of *Escherichia*-*Shigella* in ticks fed on *E. coli*-immunized mice. This result suggests that bacterial community modulation by anti-microbiota vaccines could impact the functional profiles associated with the tick microbiome. Considering that lysine is an essential amino acid and that the tick genome does not encode for lysine synthesis enzymes (58), it is possible that a reduction of lysine degradation by tick microbiome might result in higher levels of free lysine available for tick metabolism. This could potentially explain the higher body weight of ticks fed on *E. coli*-immunized mice compared to mock-immunized and *L. mesenteroides*-immunized mice. The limitation here is that we used pathway prediction and the availability of free lysine in the gut of ticks fed on *E. coli*-immunized mice was not experimentally tested. Further investigation is needed to examine the metabolites dynamics *in vivo* in response to bacterial community modulation by anti-*E. coli* IgM and IgG.

## Conclusions

Immunization with a live *E. coli* vaccine elicited an anti-*E. coli* IgM and IgG response that reduced the abundance of the keystone taxon *Escherichia-Shigella* in the tick microbiome. The results suggest that host Abs bind and kill *Escherichia-Shigella* bacteria in the tick microbiome, a conclusion supported by two evidences: (*i*) a negative correlation between the levels of both anti-*E. coli* IgM and IgG and the relative abundance of *Escherichia-Shigella* in the tick microbiome and (*ii*) the binding of anti-*E. coli* IgM and IgG to *E. coli ex vivo*. Thus, anti-tick microbiota vaccines can be used to target specific taxa within the tick microbiota through host antibodies (59). We also showed that tick engorgement, microbiome bacterial diversity and microbial community structure can be disturbed by vaccination with *E. coli* highlighting the important role that keystone microbiota bacteria have in tick performance and microbiome. Changes in the abundance of predicted enzymes and pathways suggest that the scope of anti-tick microbiota vaccine is not limited to the modulation of the tick microbiome at the taxonomic level, but it may also regulate the functions associated with the microbiome. As in our previous study (15), here we validated the use of PICRUSt2 as a suitable tool to detect functional changes in the tick microbiome. In summary, targeting keystone bacteria of the tick microbiota by host Abs seems to be a suitable tool for the modulation of tick microbiome to study the role of a specific taxon in tick physiology. Anti-tick microbiota vaccine can also be a powerful tool to evaluate the functional contribution of a specific taxon in tick microbiota on pathogen colonization and transmission. These results guide precise interventions for the control of tick infestations and pathogen infection/transmission (59).

## Data Availability Statement

The datasets presented in this study can be found in online repositories. The names of the repository/repositories and accession number(s) can be found below: https://www.ncbi.nlm.nih.gov/, PRJNA725498.

## Ethics Statement

The procedures were reviewed and approved by the Ethics Committee (ComEth, Anses/ENVA/UPEC), with permit number E 94 046 08.

## Author Contributions

AC-C, DO, JM, and LM-H conceived the study. LM-H, AW-C, JM, JB, and AH performed the experiments and acquired the data. DO, LM-H, SD-S, AE-P, AC-C, AH, and AW-C analyzed the data. DO, AC-C, AW-C and AE-P prepared figures and supplementary materials. LGB-H, ET-M, NV, and JF contributed reagents and other resources. AC-C, LS, LB-H, and JF supervised the work. AC-C, LM-H, AW-C, AH, and DO drafted the first version of the manuscript. All authors contributed to the article and approved the submitted version.

## Funding

UMR BIPAR is supported by the French Government’s Investissement d’Avenir program, Laboratoire d’Excellence “Integrative Biology of Emerging Infectious Diseases” (grant no. ANR-10-LABX-62-IBEID). Alejandra Wu-Chuang is supported by Programa Nacional de Becas de Postgrado en el Exterior “Don Carlos Antonio López” (grant no. 205/2018).

## Conflict of Interest

The authors declare that the research was conducted in the absence of any commercial or financial relationships that could be construed as a potential conflict of interest.

## Supplementary Material

Supplementary Figure 1 Impact of anti-microbiota vaccines on fecal microbiota of mice. **(A)** Comparison of ASV richness between *E. coli*-immunized (red) and mock-immunized mice. **(B)** PCoA on Bray Curtis dissimilarity from *E. coli*-immunized (red) and mock-immunized mice (green) at d0 (T0), d30 (T2) and d46 (TF), compared by permutational (Adonis) test with 999 permutations. **(C)** Dendrogram heatmap on the taxonomic profile from all the samples in both vaccination groups at the three different time points. **(D)** The relative abundance of the top 20 most abundant taxa, including *Escherichia-Shigella* (red asterisk) is displayed.

Supplementary Figure 2 Rarefaction curves estimated from reads obtained for ticks and mice. **(A)** Rarefaction curves for unfed tick samples (light orange), and samples of ticks fed on mock-immunized (dark orange), *E. coli*-immunized (dark blue) and *L. mesenteroides*-immunized (light blue) mice are displayed. **(B)** Rarefaction curves for fecal samples in *E. coli*-immunized (dark blue) and mock-immunized mice are displayed.

Supplementary Figure 3 Immunocytochemistry of *E. coli* and *L. mesenteroides* using sera of immunized mice. Fixed *E. coli* **(A)** and *L. mesenteroides* **(B)** were stained with pooled sera of mice immunized with a live *E. coli* vaccine (*Escherichia*), live *L*. *mesenteroides* vaccine (*Leuconostoc*) or mock vaccine (PBS). Examples of positive reaction are displayed (white arrows in inserts). Alexa fluor 488 conjugated anti-mouse antibody specific to the isotypes IgM and IgG were used as a secondary antibody. Negative control staining (Control) was performed using only the secondary antibody. Blue color indicates the nuclei visualized by 4’,6-diamidino-2-phenylindole (DAPI). Images were obtained using 63X magnification and digital zoom. Scale bars are 2µm.

Supplementary Figure 4 Antibody response of mice vaccinated with live *E. coli* or *L. mesenteroides.* The levels of IgM and IgG specific to **(A)** *E. coli* and **(B)** *L. mesenteroides* proteins were measured by semi-quantitative ELISA in sera of mice immunized with *L. mesenteroides* (blue) and *E. coli* (red), respectively, at different time points, d0, d14, d30 and d46. Antibody levels of bacteria-immunized mice were compared with those of mock-immunized (green, PBS) mice. Means and standard error values are shown. Results were compared by two-way ANOVA with Bonferroni test applied for comparisons between control and immunized mice. (**p* < 0.05, ***p* < 0.001, ****p* < 0.0001; ns-not significant; 1 experiment, n = 12 mice and three technical replicates per sample.

Supplementary Figure 5 A schematic representation of the co-occurring microbial taxa in the microbiome of ticks fed on mock-immunized **(A)**, *L. mesenteroides*-immunized **(B)** and *E. coli*-immunized **(C)** mice. Circles (nodes) are bacterial genera and edges the co-occurrence between taxa. Equal colors mean clusters of taxa that co-occur more frequently among them than with other taxa. The size of the circles is proportional to the eigencentrality of each taxon in the resulting network. The nodes *Escherichia*-*Shigella* (red) and *Leuconostoc* (cyan) were identified and labelled (lighting symbol).

Supplementary Table 1 Predicted genes (KO) with differential abundance in the ticks that fed on mock-immunized mice compared to unfed ticks.

Supplementary Table 2 Predicted genes (KO) with differential abundance in the ticks that fed on *L. mesenteroides*-immunized compared to mock-immunized mice.

Supplementary Table 3 Predicted genes (KO) with differential abundance in the ticks that fed on *E. coli*-immunized compared to mock-immunized mice.

Supplementary Table 4 Pathways with differential abundance in the ticks that fed on mock-immunized mice compared to unfed ticks.

Supplementary Table 5 Pathways with differential abundance in the ticks that fed on *L. mesenteroides*-immunized compared to mock-immunized mice.

Supplementary Table 6 Pathways with differential abundance in the ticks that fed on *E. coli*-immunized compared to mock-immunized mice.
