# Supplementary figures and images for: Anti-Microbiota Vaccines Modulate the Tick Microbiome in a Taxon-Specific Manner
Source: Front Immunol. 2021 Jul 12;12:704621. doi: 10.3389/fimmu.2021.704621 (PMC8312226; doi:10.3389/fimmu.2021.704621)

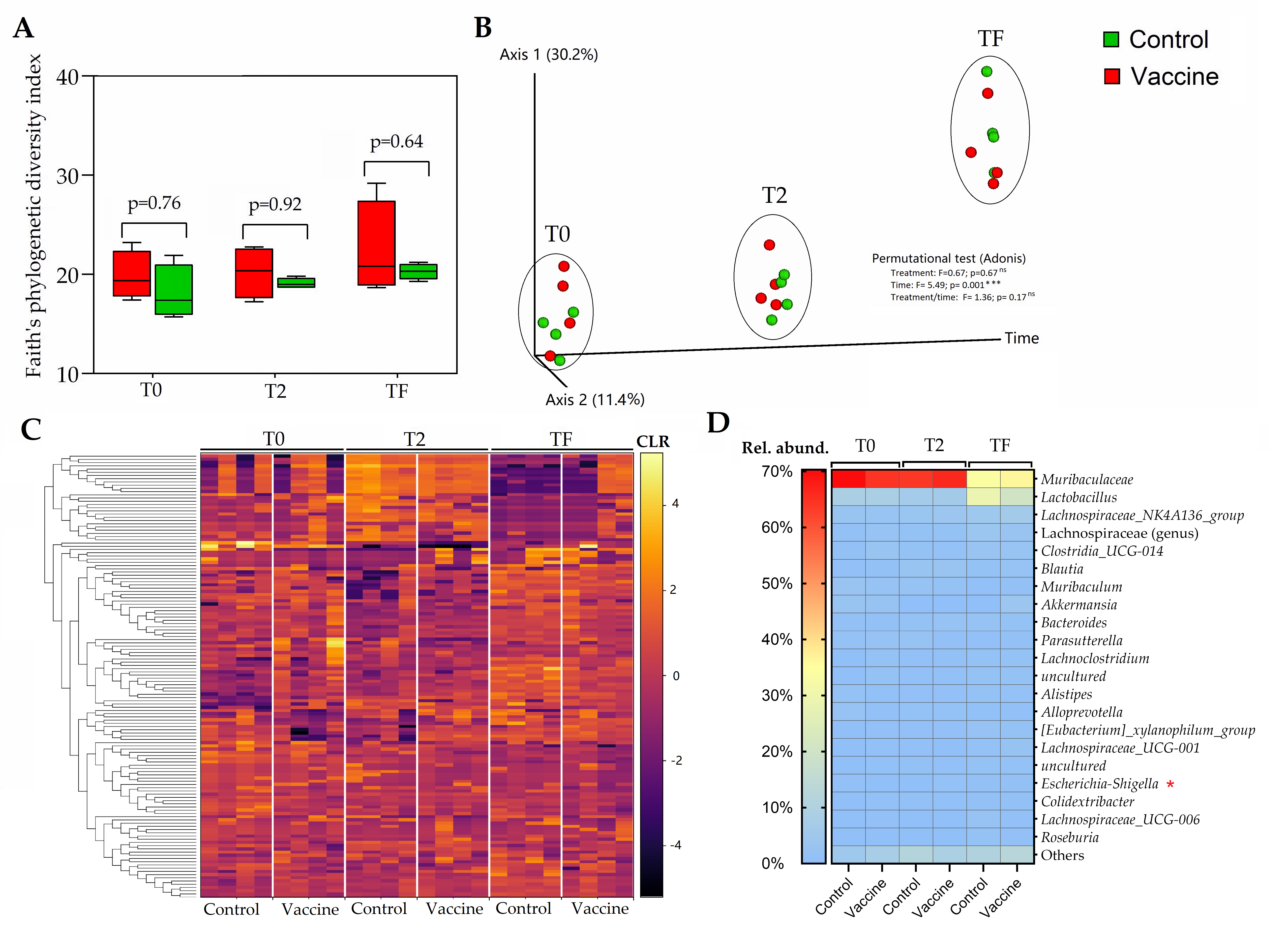

Supplement: Supplementary Figure 1 — Impact of anti-microbiota vaccines on fecal microbiota of mice. (A) Comparison of ASV richness between E. coli-immunized (red) and mock-immunized mice. (B) PCoA on Bray Curtis dissimilarity from E. coli-immunized (red) and mock-immunized mice (green) at d0 (T0), d30 (T2) and d46 (TF), compared by permutational (Adonis) test with 999 permutations. (C) Dendrogram heatmap on the taxonomic profile from all the samples in both vaccination groups at the three different time points. (D) The relative abundance of the top 20 most abundant taxa, including Escherichia-Shigella (red asterisk) is displayed. [file Image_1.tif]

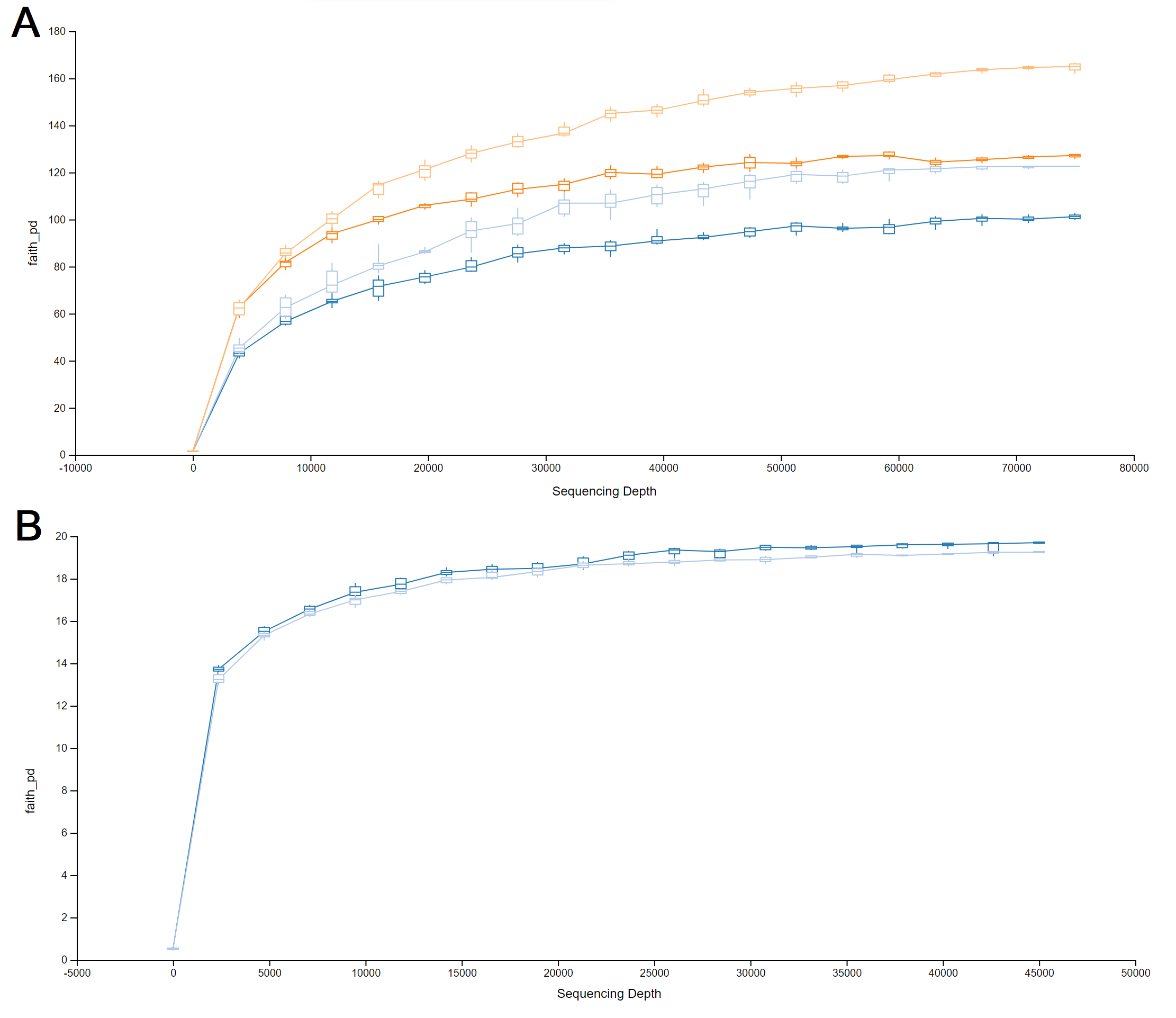

Supplement: Supplementary Figure 2 — Rarefaction curves estimated from reads obtained for ticks and mice. (A) Rarefaction curves for unfed tick samples (light orange), and samples of ticks fed on mock-immunized (dark orange), E. coli-immunized (dark blue) and L. mesenteroides-immunized (light blue) mice are displayed. (B) Rarefaction curves for fecal samples in E. coli-immunized (dark blue) and mock-immunized mice are displayed. [file Image_2.tif]

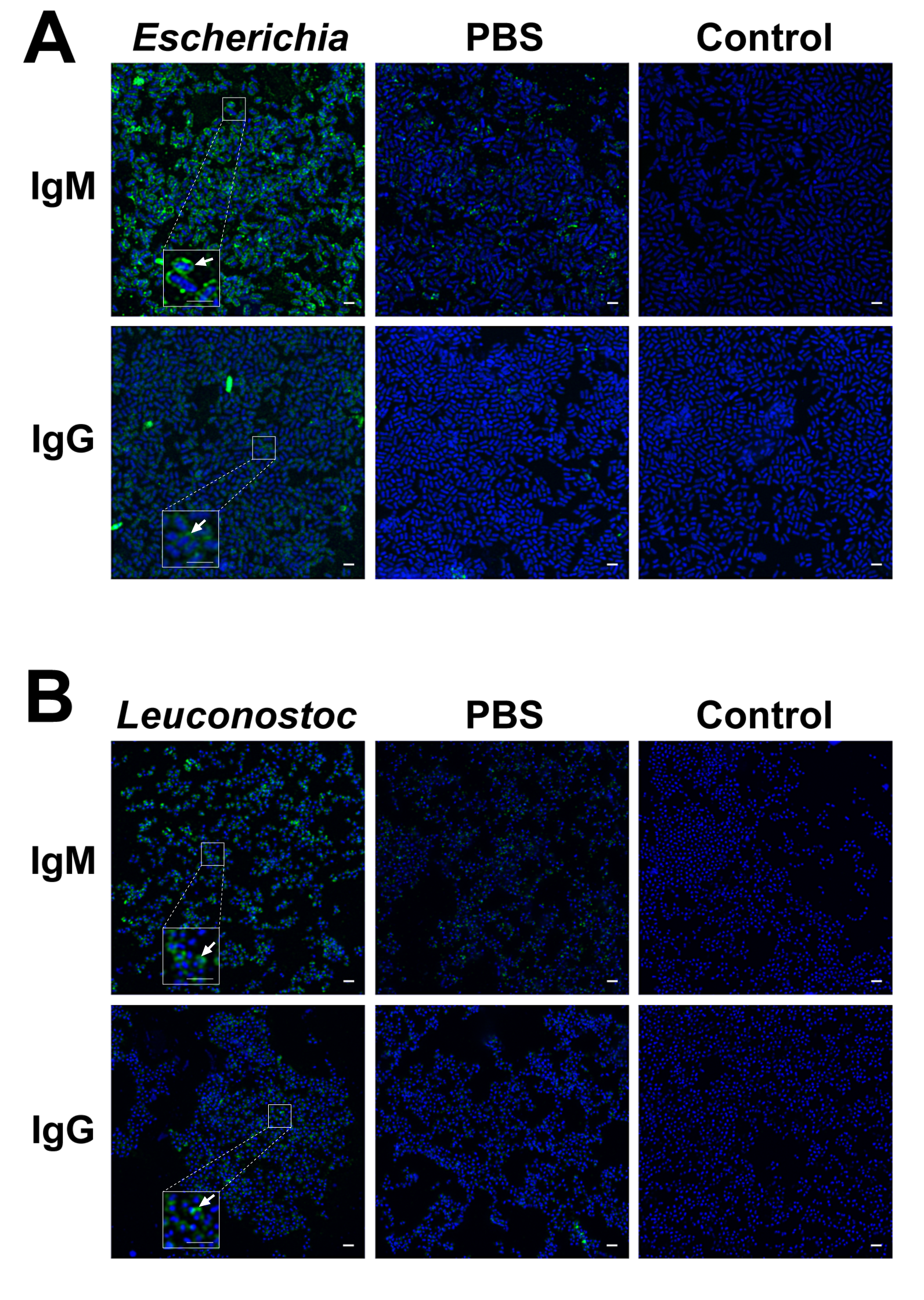

Supplement: Supplementary Figure 3 — Immunocytochemistry of E. coli and L. mesenteroides using sera of immunized mice. Fixed E. coli (A) and L. mesenteroides (B) were stained with pooled sera of mice immunized with a live E. coli vaccine (Escherichia), live L. mesenteroides vaccine (Leuconostoc) or mock vaccine (PBS). Examples of positive reaction are displayed (white arrows in inserts). Alexa fluor 488 conjugated anti-mouse antibody specific to the isotypes IgM and IgG were used as a secondary antibody. Negative control staining (Control) was performed using only the secondary antibody. Blue color indicates the nuclei visualized by 4’,6-diamidino-2-phenylindole (DAPI). Images were obtained using 63X magnification and digital zoom. Scale bars are 2µm. [file Image_3.tif]

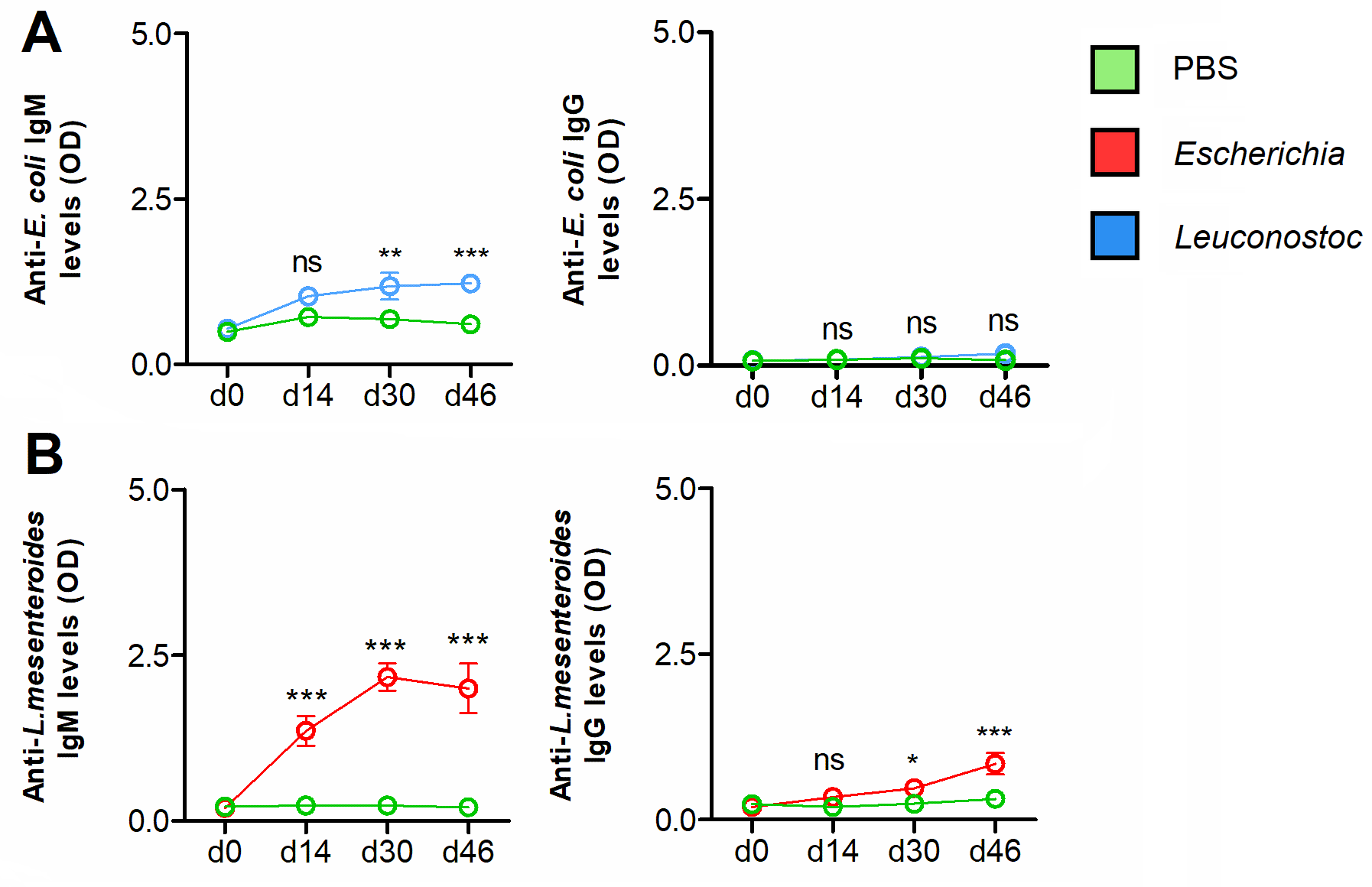

Supplement: Supplementary Figure 4 — Antibody response of mice vaccinated with live E. coli or L. mesenteroides. The levels of IgM and IgG specific to (A) E. coli and (B) L. mesenteroides proteins were measured by semi-quantitative ELISA in sera of mice immunized with L. mesenteroides (blue) and E. coli (red), respectively, at different time points, d0, d14, d30 and d46. Antibody levels of bacteria-immunized mice were compared with those of mock-immunized (green, PBS) mice. Means and standard error values are shown. Results were compared by two-way ANOVA with Bonferroni test applied for comparisons between control and immunized mice. (*p < 0.05, **p < 0.001, ***p < 0.0001; ns-not significant; 1 experiment, n = 12 mice and three technical replicates per sample. [file Image_4.tif]

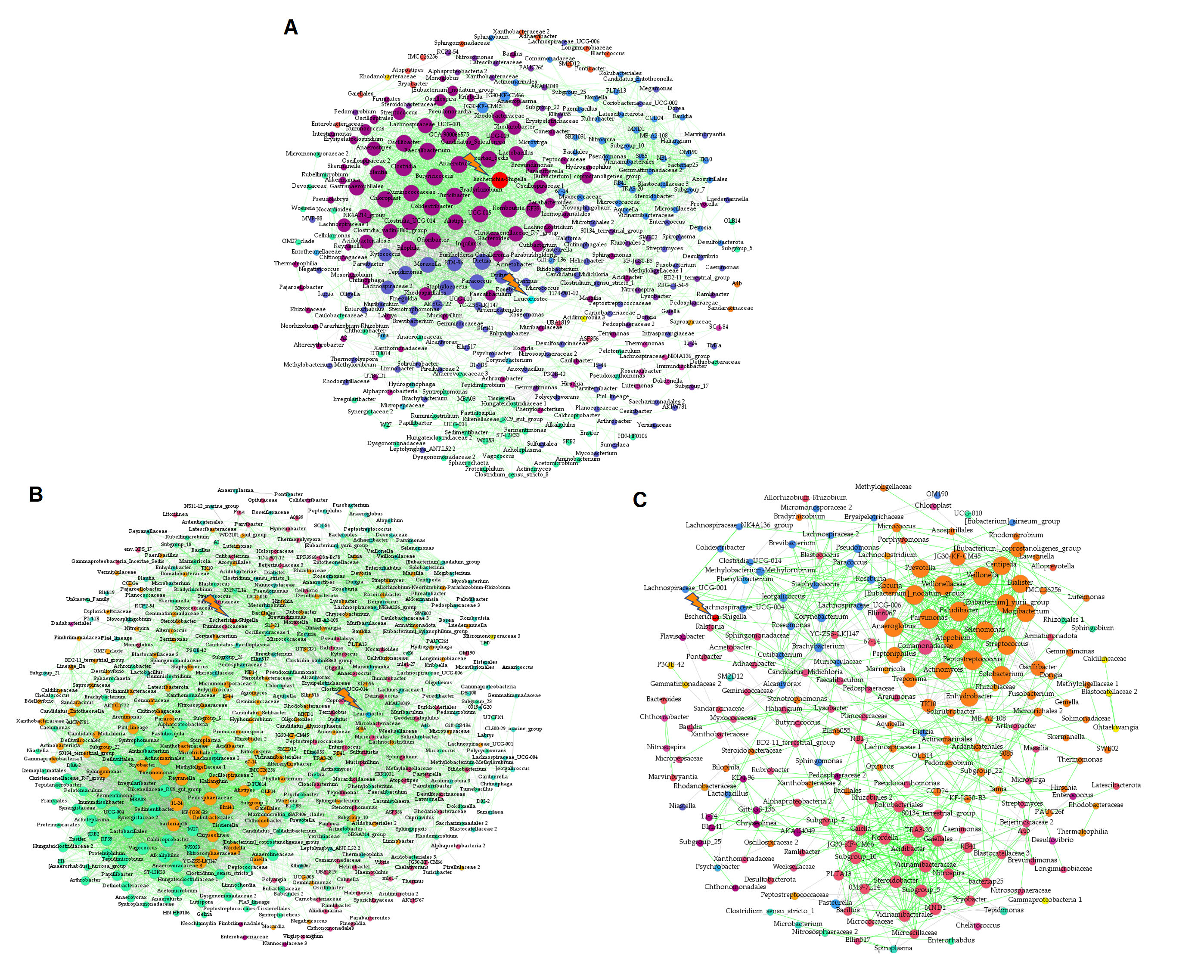

Supplement: Supplementary Figure 5 — A schematic representation of the co-occurring microbial taxa in the microbiome of ticks fed on mock-immunized (A), L. mesenteroides-immunized (B) and E. coli-immunized (C) mice. Circles (nodes) are bacterial genera and edges the co-occurrence between taxa. Equal colors mean clusters of taxa that co-occur more frequently among them than with other taxa. The size of the circles is proportional to the eigencentrality of each taxon in the resulting network. The nodes Escherichia-Shigella (red) and Leuconostoc (cyan) were identified and labelled (lighting symbol). [file Image_5.tif]
